# An autosomal dominant cataract locus mapped to 19q13-qter in a Chinese family

**Published:** 2011-01-25

**Authors:** Rui Zhao, Yonjia Yang, Xinyu He, Zheng Liu, Pin Wang, Lijun Zhou, Jinsong Tang, Wei Xu, Liping Li, Yimin Zhu

**Affiliations:** 1 The Laboratory of Genetics And Metabolism, Hunan Children’s Research Institute, Hunan Children’s Hospital, University of South China, Changsha, China; 2 School of Biology, Georgia Institute of Technology, Atlanta, GA; 3 Department Of Ophtalmology, Hunan Children’s Hospital, University of South China, Changsha, China; 4 The Laboratory of Basic Medicine, Hunan Children’s Research Institute, Hunan Children’s Hospital, University of South China, Changsha, China; 5 Institute of Mental Health,The Second Xiangya Hospital, Central South University, Changsha, China; 6 The First Hospital of Hunan Province, Changsha, China; 7 Department of Emergency, Hunan Children’s Hospital, University of South China,Changsha, China

## Abstract

**Purpose:**

The aim of this study was to map the disease locus of autosomal dominant cataracts (ADC) in a Chinese family.

**Methods:**

A four-generation family with multiple individuals affected by ADC was investigated. Genomic DNA was collected from 22 family members. A gene-scan at known candidate ADC loci was performed. To achieve fine-mapping we genotyped fourteen STR markers at the critical region of 19q.

The two-point logarithm of odds (LOD) score was calculated using Linkage Software Package Version 5.1 for linkage analysis. The haplotype was constructed using Cyrillic software.

**Results:**

Ten members of this Chinese family were affected by nuclear cataracts. Initially,  linkage analysis revealed a significant LOD score of 3.82 at the STR marker D19S418. Subsequently, after refine-marker analysis, a maximum LOD score (Z_max_=4.25) was obtained at the D19S877 (θ=0). Haplotype analysis also confirmed the locus and further narrowed it down to a critical interval from the marker D19S924 to the 19qter.

**Conclusions:**

We have successfully mapped an ADC locus to 19q13-qter. Previous studies have identified three cataract loci on 19q; however, we found no overlap between the locus of this study and any of the previously identified loci. We therefore suggest that the 19q13-qter locus in this family is a new locus for ADC.

## Introduction

Cataract is a disease associated with opacity of the ocular lens. Along with the cornea, the lens is the organ that transmits and refracts light. Also, the lens is the only tissue in mammals capable of accurately focusing light onto the retina through a process called accommodation [1]. Therefore, cataract-induced opacity of the lens results in dramatic visual impairment, delayed visual developmental and blindness in humans. For example, cataracts have caused one million cases of childhood blindness in Asia [2].

The phenotype (or spectrum of morphology) for cataracts is variable and complex. However, according to the location of the opacification the cataract can be categorized as embryonic nucleus, fetal nucleus, cortex, or pole. Of these types, opacification affecting the nucleus is the most common [2].

According to the age of onset, cataracts can be categorized as: (1) congenital cataract:  onset age is less than one year; (2) juvenile: onset-age is from 1 to 10 years; (3) presenile cataract: onset-age is above 10 and up to to 45 years; (4) age-related cataract: above 45 years. Of these, about 25 percent of the congenital and juvenile cataracts are believed to be inherited [1,3].

Inherited cataracts, transmitted with the inheritance of autosomal dominant (AD), autosomal recessive (AR) and X-linked (XL) genes [4], are a genetically heterogeneous eye-disorder. Renwick and Lawler first described the inherited cataract cosegregated with the Duffy blood group locus (OMIM 110700) in 1963  [5]; the Duffy locus was then further assigned to chromosome 1 [6] and  since then there have been at least 40 loci for human chromosomes linked to inherited isolated (or non-syndromic) cataracts, including 26 loci with a clear disease-causing gene. The remaining 14 loci, including 1p36, 1p34-p36, 1q25-q13, 2p24, 2p21, 3p22-p24.2 (AR), 9q13-q22 (AR), 15q21-q22, 17q24, 19q13, 13q13.4 (AR), 20p12.2-q12(p12.2-p11.23), Xp22, and 3q22.3-q25.2, have been regarded as isolated cataracts where no disease-causing gene could be clearly identified [1,7-9].

Here, we report on a Chinese family affected by inherited, isolated nuclear cataracts. In our study, we present evidence linking the family’s disorder to chromosome 19q. Refined analysis of STR-linkage and haplotype construction have further confirmed the autosomal dominant cataracts locus and narrowed it to 19q13-qter. Previous studies have identified three isolated cataract loci on 19q; these include the 19q12-q13.1 (autosomal recessive (AR) gene, between markers D19S928 and D19S425) [10], the 19q13.1-q13.33 (AD)  gene, between markers D19S220 and D19S902) [11] and *LIM2* (AR) [12]. However, we have found no overlap between the locus in this study or with any of the previously identified isolated cataracts loci on 19q. Thus, we suggest that the 19q13-qter (from marker D19S924 to qter) locus is a new ADC locus.

## Methods

### Family description and DNA isolation

**Figure 1:**
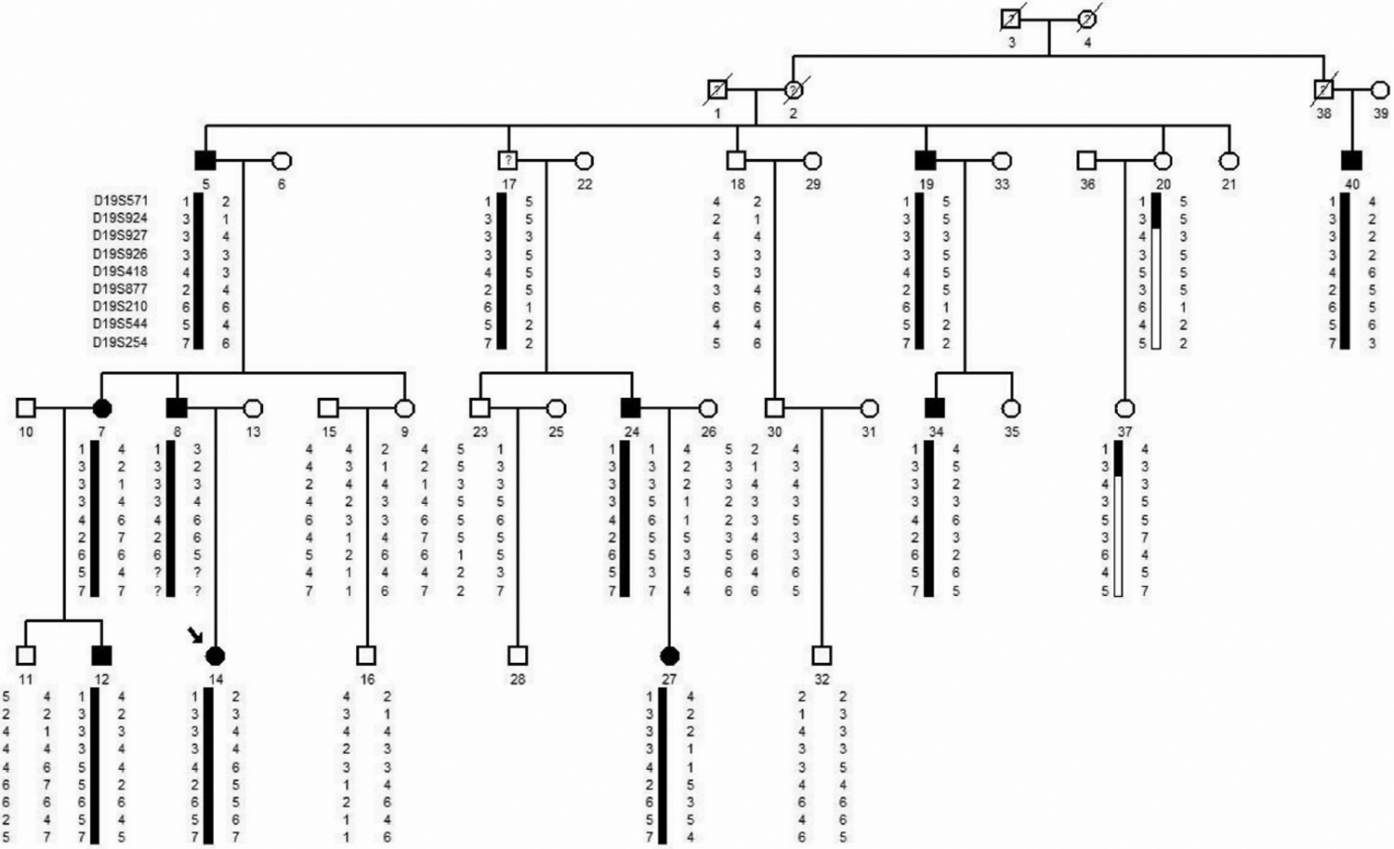
Pedigree of this study. The affected individuals are denoted with solid symbols. The haplotypes in the critical region on chromosome 19q were indicated below each individual where linkage was studied. A question mark in the squares or circles indicates that the affected status is unknown.

A four-generation family with inherited isolated cataract was investigated (Figure 1). Twenty-two members of the family participated in this study, including 10 affected and 12 unaffected individuals (Figure 1). Before sample collection, this study was approved by the Ethics Committee of the Hunan Children’s Hospital (located in Changsha City, P.R. China). The procedure of the Committee conformed to the principles of the declaration of Helsinki. Peripheral blood (from 2 to 3 ml) was collected from all 22 participating family members. The gDNA was isolated by standard procedure using the phenol/chloroform extraction method. All specimens were quantified by spectrophotometry and diluted to 25 ng/μl for Polymerase Chain Reaction (PCR) amplification.

### Genotyping

Initially, a part-genome scan was performed with highly polymorphic STR markers (ABI PRISM® Linkage Mapping Set Version 2.5; Applied Biosystems, Foster City, CA) at the known ADC candidate loci, including 1p36, 1p32, 1q21-q25, 2p24, 2p12, 2q33-q35, 3q21-q22, 3q26-qter, 8q13.3, 10q25, 11q23-q24, 12q12-q14, 13q11–13, 15q21-q22, 16q22-q23, 17p13, 17q11-q12, 17q24, 19q13, 20p12.2-p11.23, 21q, and 22q. Subsequently, fourteen refining primers of short tandem repeat (STR) markers (from Marshfield Clinic Human Genetics linkage map) at 19q were synthesized with fluorescein-labeled 5′. For selected markers, multiplex PCRs were performed in a 5.0 μl reaction mixture containing 25 ng of genomic DNA, 1×PCR buffer, 100 umol of each dNTP, 3.0 mmol MgCl_2_, 80 pmol each of forward and reverse primers and 0.2 U of AmpliTaq Gold DNA polymerase. The mixture, including reaction products 1.0 μl, Liz Size Standard-500 0.2 μl and Hi-Di formylamine 9 μl, was denatured, electrophoresed and visualized on a 3130 Genetic Analyzer (Applied Biosystems). The allele size was analyzed using Genescan analysis software V3.7 and Genotyper software V3.7 (Applied Biosystems).

### Linkage calculation

Two-point linkage analyses were performed with the MLINK program of the LINKAGE package (version 5.1). The disease was an assumed autosomal dominant trait with 98% penetrance. Marker allele frequencies were set at 1/n, where n is the number of alleles observed. We assumed gene frequencies of 0.0001 and no gender-based difference in rates of recombination. Haplotype construction was then performed to define the borders of the cosegregating region using the Cyrillic program.

## Results

### Clinical description

A four-generation Chinese family (Figure 1) was investigated, in which bilateral cataracts had been diagnosed in ten individuals. The cataracts in this family were segregated to the mode of autosomal dominant. Male-to-male transmission was observed. The proband in this family is a 3.5 year-old female (ID14, Figure 1), whose onset age, as her mother recalled, was less than one-year-old. The phenotype of this proband consists of bilateral, white opacification of the embryonic nucleus. Her affected father (ID 8, Figure 1, 32-years-old), was diagnosed at the age of 17 years. His phenotype presents with bilateral, white opacification of embryonic or fetal nucleus-type, rather than cortex-type. Similar findings were seen in individuals ID5, ID7, ID24, ID27, ID19, ID34 and ID40. Nystagmus was also present in some individuals (ID5, ID24 and ID34). The visual acuity of all affected family members was damaged in a range from mild to severe. Since there was no family history of other ocular or familial abnormalities, we defined the ADC in this family as isolated. Detailed clinic data are provided in Table 1.

**Table 1:** Clinical details of the family members.

**Individual**	**Visual acuity**	**Age (years)**	**Nuclear cataracts**	**Nystagmus**	**Other findings**
5	L0.2 R0.2	65	pulverulent with dots	yes	
7	L0.4R0.2	40	nucleus dots	no	freckles
8	L0.1R0.3	34	nucleus dots	no	mild freckles
12	L0.4R 0.6	15	embryonic nucleus dots	no	
14	NA	3	embryonic nucleus dots	no	
24	NA	33	nucleus dots	yes	
27	L0.3R.04	6	nucleus dots	no	
19	NA	56	pulverulent with dots	no	
34	NA	29	nucleus dots	Yes (mild)	
40	NA	37	nucleus dots	NA	mental retardation

NA indicates the data not available.

### Linkage analysis

Twenty-two members of the family, including ten affected individuals and twelve unaffected individuals, were genotyped with microsatellite markers at the following 22 ADC loci: 1p36, 1p32, 1q21-q25, 2p24, 2p12, 2q33-q35, 3q21-q22, 3q26-qter, 8q13.3, 10q25, 11q23-q24, 12q12-q14, 13q11–13, 15q21-q22, 16q22-q23, 17p13, 17q11-q12, 17q24, 19q13, 20p12.2-p11.23, 21q and 22q. Using two-point linkage analysis, we excluded all the loci mentioned above (data not shown) except for 19q. In the 19q locus a significant logarithm of odds (LOD) score was initially found at the marker D19S418 (LOD=3.82, at θ=0). To confirm this finding, we subsequently synthesized fourteen pairs of refining primers (some of the STRs are low-informative). The following genotyping and linkage analysis of the refining markers showed a maximum LOD score of 4.25 and 3.99 at markers D19S877 and D19S544 (θ=0), respectively (Table 2). Ensuing haplotype construction also confirmed the 19q ADC locus. On two unaffected individuals (ID. 37 and ID. 20), a critical recombination event was found (Figure 1). This recombination event further defined the disease gene interval proximal to the marker D19S924, while, the distal part of chromosome 19q were found in no other recombination in this family group (Figure 1). Individual #37 (Figure 1) was a 16 year-old-girl. Her unaffected status was ascertained by two independent ophthalmologists. Her father, (individual #20), was an important family member in terms of defining the locus border; he was one of the unaffected group. Detailed physical examination of this man revealed signs of adolescent leukotrichia; however, we detected no any sign of cataracts with the slit lamp examination performed by an experienced ophthalmologist. In brief, we have assigned the ADC locus in this family to an interval of 4.9Mb, from the D19S924 to 19qter.

**Table 2:** Two-point LOD score for microsatellite markers on human chromosome 19q in the Chinese ADC family.

** **	** **	** **	**Lod score at θ=**					** **
**STR**	**Physical position (Mb)**	**Genetic position (cM)**	**0.00**	**0.10**	**0.20**	**0.30**	**0.40**	**Z_max_**
D19S924	54.1	88.9	−5.13	1.25	1.12	0.80	0.39	1.25
D19S927	54.2	89.7	2.39	1.90	1.38	0.83	0.31	2.39
D19S926	55.4	78.1	1.47	1.18	0.87	0.56	0.26	1.47
D19S418	55.5	92.6	3.82	3.14	2.39	1.57	0.68	3.82
D19S877	55.8	95.3	4.25	3.46	2.61	1.67	0.68	4.25
D19S210	57.0	100	1.03	0.82	0.60	0.37	0.16	1.03
D19S544	57.1	100	3.99	3.26	2.47	1.60	0.69	3.99
D19S254	57.6	100.6	2.36	1.82	1.32	0.81	0.30	2.36

## Discussion

In this study, we report on a Chinese family affected by inherited autosomal dominant nuclear cataract. We extracted gDNA samples from 22 family members and performed linkage analysis of the known-candidate ADC loci using the STR markers. The results excluded all known loci except for 19q. A maximum LOD SCORE of 4.25 (Table 2) was obtained at the marker D19S877 (at θ=0). Haplotype analysis identified the upper border of the disease gene in this family as D19S924. In brief, we have mapped the ADC locus to 19q13-qter, from the marker D19S924 to the distal-end of chromosome 19q (Figure 1).

**Figure 2:**
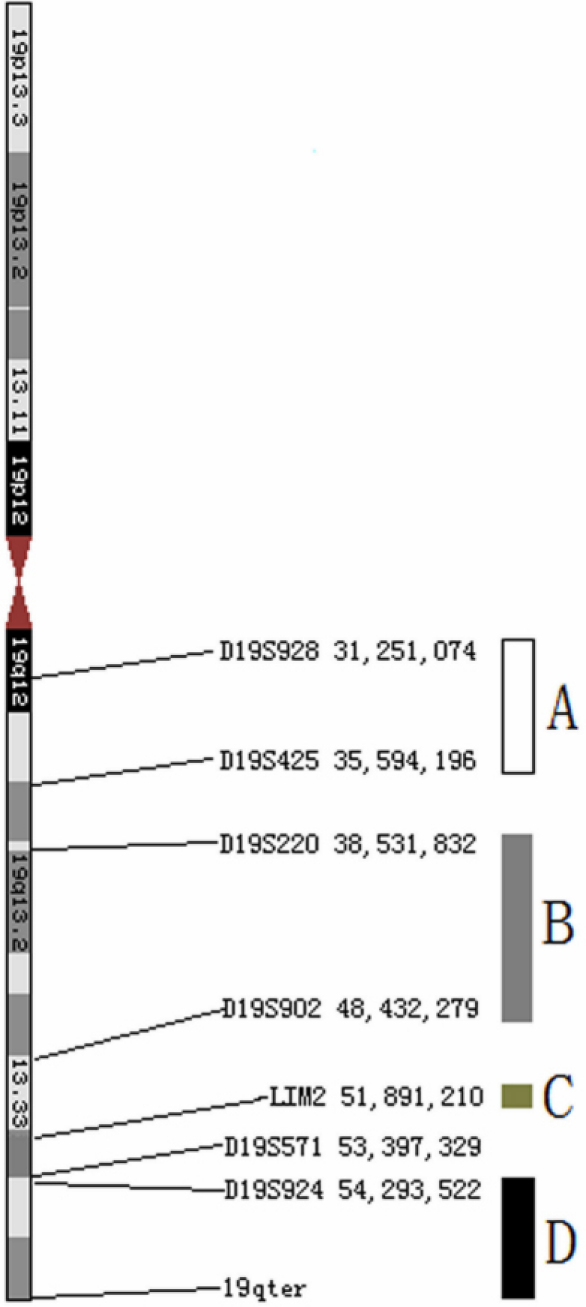
The isolated cataract loci on chromosome 19q. “A” shows the autosomal recessive cataracts locus in a Pakistani family described by Pras et al. [10]; “B” shows the autosomal dominant cataracts locus in a four-generation American family described by Bateman et al. [11]; “C” shows the location of the *LIM2* gene, witch causes autosomal recessive presenile cataract in an inbred Iraqi-Jewish family, described by Pras et al. [12] “D” shows the autosomal dominant cataracts locus identified in this study.

Cataract is a clinically and genetically heterogeneous disease. The same phenotype of cataract can be caused by different gene-mutations or by different gene loci [13]. Up to now there have been at least 41 genetic loci on the human genome (which have been well documented by Hejtmancik et al. [1,7,8]). Previous investigations have identified three loci on 19q for isolated cataract, including an autosomal recessive cataract (ARC) locus [10], an ADC locus [11] and *LIM2*—an identified ARC gene [12]. In 2004, Riazuddin et al. [10] described an ARC locus at 19q12-q13, between the markers D19S928 and D19S425 (a 4.3 MB interval) in a consanguineous Pakistani family (Figure 2). The phenotype in this Pakistani family was nuclear cataract, thus sharing some similarities with the phenotype discussed in this study. In 2006, Bateman et al. [11] identified a new locus for ADC on 19q13 between the markers D19S220 and D19S902 in one four-generation American family. The phenotype in the American family was unclear. However, as the authors described, after cataract extraction one individual retained white and vacuolated opacities in the cortical-region—a condition that can probably be classified as total cataract. In an inbred Iraqi-Jewish family, Pras et al. [12] illustrated the autosomal recessive presenile cataract caused by a missense mutation in the *LIM2* gene. Therefore, the chromosome 19q  is perhaps a hotspot for  cataract pathogenesis. To make the 19q cataract loci clear, we have illustrated them (Figure 2) according their physical position (the distance was from the UCSC Genome Browser). Figure 2 shows that the four cataract loci, including the locus from this study, were interdependent. Even though the *LIM2* gene is near to the locus in this family, the physical distance between them is at least 2.4 Mb.

In summary, we have mapped an autosomal dominant nuclear cataract locus to 19q13–19qter. From the linkage results and the position-analysis given above, we suggest that the 19q13-qter (from the marker D19S924 to qter) locus is a new ADC locus. Further study is needed to identify the disease-causing gene and provide new insights into the molecular mechanisms of the cataractogenesis occurring in this family.
